# Effects of a low-dose combination therapy of zoledronate and dexamethasone on post-tooth extraction socket healing: a pre-clinical study in mice

**DOI:** 10.1590/1678-7757-2024-0574

**Published:** 2025-07-07

**Authors:** Ana Julia Moreno BARRETO, Claudia Cristina BIGUETTI, Raquel Barroso Parra da SILVA, Letícia Citelli CONTI, Rafael Carneiro ORTIZ, Alaide GONÇALVES, Edilson ERVOLINO, Antonio Hernandes CHAVES-NETO, Mariza Akemi MATSUMOTO

**Affiliations:** 1 Universidade Estadual Paulista Faculdade de Odontologia de Araçatuba Departamento de Ciências Básicas Araçatuba SP Brasil Universidade Estadual Paulista (UNESP), Faculdade de Odontologia de Araçatuba, Departamento de Ciências Básicas, Araçatuba, SP, Brasil.; 2 University of Texas at Rio Grande Valley School of Podiatric Medicine Harlingen United States University of Texas at Rio Grande Valley, School of Podiatric Medicine, UTRGV, Harlingen, United States.; 3 Universidade Estadual do Norte do Paraná Jacarezinho Paraná Brasil Universidade Estadual do Norte do Paraná (UENP), Jacarezinho, Paraná, Brasil.; 4 Universidade de São Paulo Faculdade de Odontologia de Bauru Departamento de Ciências Biológicas Bauru SP Brasil Universidade de São Paulo, Faculdade de Odontologia de Bauru, Departamento de Ciências Biológicas, Bauru, SP, Brasil.

**Keywords:** Bone, Bisphosphonate, Glucocorticoid, Mice

## Abstract

Pharmaceutical agents targeting distinct therapeutic pathways can differently influence bone metabolism, notably exemplified by nitrogen-containing bisphosphonates and glucocorticoids.

**Objective:**

This investigation sought to elucidate the effects of a low-dose administration of zoledronate (ZL) and dexamethasone (DX) on post-tooth extraction sockets healing in a murine model.

**Methodology:**

In total, 40 young male C57BL/6J mice were assigned to four distinct groups by weight-stratified randomization: Control (C) - 0.9% saline solution, ZL - 0.05 mg/kg ZL, DX - 5 mg/kg DX, and ZL+DX - combined regimen of 0.05 mg/kg ZL and 5 mg/kg DX. All substances were intraperitoneally delivered on a weekly basis from four weeks before right upper incisor extraction and up to seven and 30 days after it, when blood was collected for biochemical analysis of bone markers and the maxillae were removed and prepared for microcomputed analysis of the trabecular architecture of the healing sockets and to set histological slices to be stained with hematoxylin and eosin and immunohistochemistry for TRAP and Runx2.

**Results:**

Histopathology and microCT showed that DX administration correlated with impaired bone formation, manifesting as reduced bone volume/total volume and trabecular thickness. Conversely, ZL exposure disrupted bone viability. However, the combination of both showed enhanced maturation in bone remodeling at day 30. Notably, DX treatment notably reduced serum calcium and phosphate levels and total TRAP. Runx-2^+^ cells significantly increased in the Control group at day seven when compared to ZL and DX-ZL and at day 30 when compared to ZL.

**Conclusions:**

Our findings showed that co-administering low doses of ZL and DX in young male mice augmented the recuperative processes of their post-extraction sockets when juxtaposed with either agent in isolation. Nevertheless, further comprehensive inquiries are needed to delineate the precise underlying mechanisms in a more controlled experimental context.

## Introduction

Several factors such as aging, nutritional conditions, trauma, diseases, and medication, can disrupt bone homeostasis.^1,2^ The drugs to enhance bone repair in pathological conditions may paradoxically disrupt bone tissue, especially in the craniofacial region. While this effect depends on various factors, nitrogen-containing bisphosphonates (NBPs)^3^ and glucocorticoids (GCs) seems to constitute significant mediators in bone response efficiency.^4,5^

NBPs have been widely prescribed to manage primary or secondary metabolic bone disorders due to their potent antiresorptive properties by inhibiting osteoclast activity^6^ by disrupting the mevalonate pathway, preventing the production of isoprenoid lipids and subsequent downstream protein prenylation, which is essential to form osteoclast F-actin rings and ruffled borders.^7^ However, this mechanism can be dysregulated and promote a pathological condition, known as medication-related osteonecrosis of the jaws (MRONJ) .^3^

Several factors can be associated with an increased risk of MRONJ, including the potency of the drug, route of administration, dosage, treatment duration, and patients’ underlying conditions, such as systemic diseases, advanced age, biological sex, concomitant medications, oral health status, and other relevant factors.^3^ One of the most significant contributors to MRONJ in NBPs seems to refer to administering high doses of zoledronate (ZL). This potent bisphosphonate is frequently administered in elevated doses in oncologic therapeutic protocols,^8^ typically serving as an adjunctive therapy for various malignancies. Its principal objective involves mitigating skeletal-related events, notably bone metastases and associated pain. However, the intricate pharmacological mechanism underlying the beneficial effects of ZL can also be implicated in the etiology of MRONJ.^9^

On the other hand, GCs configure a variety of steroid hormones secreted by the adrenal glands. They play a crucial role in both health and disease by modulating the nervous and immune systems, stress responses, and the circadian rhythm.^10^ From this GC class, dexamethasone (DX), can act on various cell types by GC receptors, showing potent anti-inflammatory and immunosuppressive effects.^11^ DX is commonly prescribed for chronic inflammatory conditions, autoimmune diseases, allergies, and some respiratory diseases and to alleviate acute inflammatory symptoms, such as post-traumatic swelling and pain.^11,12^ However, long-term therapy with GCs is associated with undesired skeletal conditions, including GC-induced osteoporosis^13^ and osteonecrosis.^14,15^ DX can also directly affect bone metabolism (as in NBPs treatments) by inducing apoptosis in osteoblasts and osteocytes,^16,17^ significantly reducing osteoblast production and interstitial fluid in the osteocyte canalicular system. DX might also prolong the life span of osteoclasts, interfere with the mechanosensitive function of osteocytes, and cause other effects.^16,18^

ZL and DX drugs can have a dual role in bone metabolism as they are also prescribed for non-malignant bone conditions such as osteoporosis.^19,20^ This dual role mainly depends on dosage and administration regimen, which often are significantly lower than those to treat malignancies.^21,22^ The dose approval of ZL by the USA Food and Drug Administration was based on a multicenter comparative study that showed that a yearly intravenous infusion of 5 mg ZL was non-inferior to a daily oral dose of 5 mg risedronate to prevent and treat GC-induced osteoporosis.^8^ While this combination therapy seems to effectively prevent MRONJ, new insights on the specific dosages and combinations of NBPs and GCs may enhance our understanding of the bone biology related to MRONJ development. Note that animal models of MRONJ fail to show the same clinical and histopathological features as humans,^23^ hence the preference for “osteonecrosis of the jaws-like lesions.”^24^

In line with this trend, extensive research has investigated the heightened risk of MRONJ onset when high doses of ZL are combined with GC.^24^ However, the literature remains scarce about low dosages of ZL in this context. Thus, this study aimed to analyze the healing process of dental sockets after tooth extraction in mice treated with low doses of ZL and DX in isolation or together since individuals that show chronic inflammatory diseases and receive corticosteroids treatment may develop malignant or not osteolytic lesions that may require therapy with ZL in different dosages. Its primary objective is to assess the impact of this combined therapy on this specific process using a pre-clinical model to describe MRONJ-related disorders. Its null hypothesis is that low doses of DX combined with ZL will fail to affect post-tooth extraction dental socket in this animal model.

## Methodology

### Study design

All animal experiments complied with the ARRIVE guidelines^25^ and followed the normative regulations of Brazilian National Council for the Control of Animal Experimentation. The mice were purchased from the Central Animal Facility for Special Mice of the School of Medicine of Ribeirão Preto – University of São Paulo. This study was approved by the Institutional Animal Care and Use Committee of São Paulo State University, School of Dentistry, Araçatuba, Brazil (FOA/UNESP protocol #00262-2019). The animals were housed in the Central Animal Facility in polypropylene cages with five animals each. Their housing conditions included a controlled room temperature (RT; 22 ± 2,5^o^C), a 12-hour dark/light cycle, and a humidity range of 40-70%. The animals had access to water and pelleted food (Nuvilab CR-1, Nuvital Nutrientes S/A, Colombo, PR, Brazil) ad libitum, except for three days after the surgeries, when they were offered soft food. The animals were daily monitored by the researchers to observe their clinical conditions, signs of pain, aggression, or different behavior. To mitigate potential bias related to baseline body weight, a weight-stratified randomization procedure was implemented prior to treatment allocation. In total, 40 male C57BL/6J mice aged 12-18 weeks (mean weight: 28g) were individually weighed. The mice were rank-ordered by weight and partitioned into 10 strata with four individuals. Within each stratum, the mice were randomly assigned to one of four treatment groups (n=10 per group) by a manually generated random number sequence. This stratified randomization ensured homogeneity of body weight distribution across treatment groups. To maintain objectivity, the allocation process was conducted by a researcher who had been blinded to the experimental treatments. The groups were divided into: C – negative control, treated with 0.9% saline solution; ZL – 0.05 mg/Kg of ZL (Sigma-Aldrich, Zoledronic acid monohydrate, Darmstad, Germany); DX – 5 mg/Kg of dexamethasone (Sigma-Aldrich, Dexamethasone, Darmstad, Germany); and ZL+DX – combination of 0.05 mg/Kg ZL plus 5 mg/Kg of DX. Sample size was calculated based on OpenEpi, version 3,^26^ with a 95% confidence interval, resulting in 11 animals per group and period (Supplementary Table 1). The use of 12 animals were based on biochemical analyses.^27^ All substances were administered via intraperitoneal (IP) injections by two operators (AJMB and RBPS). The animals had received 200 μg/Kg of ZL prior to tooth extraction. Animals euthanized at the seven-day time point received 250 μg/Kg at the time of euthanasia, whereas those at the 30-day time point were treated with a total of 400 μg/Kg. Despite cumulative dosages, no animal showed kidney or liver injuries (Data not shown).

**Table 1:** Means and standard deviations obtained from the quantification of the following mCT parameters from healing sockets on day 30: BV/TV(%), Tb.Th (mm), Tb.Sp (mm), and Tb.N (mm).

Parameters	C	DX	ZL	DX+ZL
BV/TV	89.37±2.68^b^	72.44±8.58^a^	95.66±1.98^b^	87.62±6.09^b^
Tb.Th	0.61±0.09^a^	0.33±0.01^b^	0.54±0.06^a^	0.51±0.05^a^
Tb.Sp	0.14±0.04^a^	0.29±0.18^a^	0.22±0.30^a^	0.16±0.04^a^
Tb.N	1.57±0.14^a^	1.9±0.10^a^	1.75±0.16^a^	1.79±0.14^a^

*Different letters indicate significant differences between groups considering the same time point (p<0.05). (C=control, DX=dexamethasone, ZL=zoledronate, ZL + DX=zoledronate + dexamethasone, BV/TV=bone volume fraction, Tb.Th=trabecular thickness, Tb.Sp=trabecular spacing, Tb.N=trabecular number).

### Tooth-extraction procedure and sample collection

Overall, 48 hours after the fourth dose of saline, ZL, DX, and concomitant doses of ZL and DX, all animals underwent tooth extraction, as previously described (Mahmoud 2021, Biguetti 2023).^28,29^ They were anesthetized with a combination of ketamine (80-100 mg/Kg, Dopalen^®^, Agibrands do Brasil, Ltda.) and xylazine (10 mg/Kg, Anasedan^®^, Agibrands do Brasil, Ltda.). Intraoral antisepsis was performed using a topical 1% polyvinylpyrrolidone prior to the procedures to extract their right upper incisor. First, the attached gingiva was detached using a micro scFALer, and the tooth was dislodged with a #5 exploratory probe and removed using a micro forceps (Fine Surgical Instruments for Research, Canada). A careful visual inspection was performed to confirm if the apical dental germ was attached to the tooth (if so, it was removed during surgery).

After the tooth extraction, a soft standard diet was offered for three days to minimize pain and prevent unnecessary local trauma. Euthanasia was scheduled for seven- and 30-days post-tooth extraction under deep anesthesia, followed by decapitation to collect the maxillae. Additionally, at day 30, approximately 2 ml of blood were collected from each animal in heparinized tubes. The collected blood samples were immediately transferred and centrifugated at 1000 × g at 4^o^C for 10 minutes to obtain plasma, which was stored in an ultra-freezer at −80^o^C until the biochemical analysis. According to a previous study from our research group, the maxillae were fixed in buffered 10% formalin and immersed in 70% alcohol 48 hours late for microcomputed tomography scanning (µCT).^30^

### µCT scan acquisition

Only the maxillae at 30 days were µCT scanned (SkyScan 1174 System, Kontich, Belgium) at 50kV, 800 µA, with a 0.5-mm aluminum filter, 180^o^rotation, 1-range degree exposition, and 14-µm resolution smoothing 4, ring artifact correction 4, and beam hardening correction of 15% at Faculdade de Odontologia de Araçatuba. Then, the images were reconstructed on NRecon (version 1.6.9.8, SkyScan 2005-2012, Bruker microCT^®^), realigned in Data Viewer (version 1.5.1.2, SkyScan 2004-2011, Bruker microCT^®^) in the sagittal position for the quantitative and qualitative analysis of bone microarchitecture formed inside the dental sockets using CTAnalyzer (version 1.14.4.1, SkyScan 2003-2011, 2012-2014 Bruker microCT^®^) to quantify trabecular parameters. The region of interest encompassed a 3-mm length and a 1-mm diameter cylinder comprising the long axis of the dental socket from the coronal to the apical region. The parameters used to analyze trabecular microarchitecture were bone volume fraction (BV/TV%), trabecular thickness (Tb.Th, mm), separation (Tb.Sp, mm), and number (Tb.N, mm), as per Bouxsein, et al. ^31^ (2010). Analysis was performed by a single operator (CCB).

### Descriptive histological analysis and histomorphometry

After the µCT scan, the maxillae were demineralized in buffered 4% ethylenediaminetetraacetic acid, pH 7.0 (Merck, Darmstadt, Germany), for approximately 20 days after confirmation. They were then washed in running water before being dehydrated in alcohol, cleared in xylene, and embedded in paraffin (Synth, São Paulo, Brazil). Histological slices with a thickness of 5 µm were obtained from coronal sections of the sockets and stained with hematoxylin and eosin (HE).

Descriptive histological analyses of the healing sockets were performed using HE-stained slices by two calibrated observers (AJMB and MAM) who were blinded to the groups at both periods. Analysis considered parameters such as granulation tissue, bone maturing, bone sequestrum, biofilm, and inflammation. The quantitative analysis was performed after the capture of 10 fields of each histological slide in the middle third of the sockets under 100× magnification using a photomicroscope AxioCam^®^ (Carl Zeiss, Göttingen, Germany) coupled with an optical microscope (AxioLab^®^) connected to the computer with an image software (Axiovision 4.8.2^®^ Carl Zeiss). A120-point grid was built over each captured field on ImageJ (Version 1.51, National Institutes of Health, Bethesda, Maryland, EUA) using horizontal and vertical lines. When the intersection of the horizontal and vertical lines were detected over one of the chosen parameters (osteoblasts, adhered osteoclasts, detached osteoclasts, fibroblasts, blood vessels, bone matrix, collagen fibers, inflammatory infiltrate, mononuclear leukocytes, and polymorphonuclear leukocytes), they were individually counted. The obtained values of each parameter were calculated by area density in % that was obtained by calculating the proportion of points for a particular structure in relation to the total number of points (100%) in the grid.

### Immunohistochemistry and immunostaining analysis of bone markers

To detect TRAP and Runx-2 positive cells, 5-µm histological slices from the coronal sections of the sockets were treated as previously described.^32^ Slides were incubated overnight at RT with the polyclonal primary antibodies anti-TRAP (SC#30832, Santa Cruz Biotechnology, Carpinteria, USA) and anti-RUNX-2 (SC#8566, Santa Cruz Biotechnology, Carpinteria, USA) at a concentration of 1:200 for both. Then, the slices were incubated with the anti-goat immune-enzyme polymer method (ImmPRESS^®^ HRP Horse Anti-Goat IgG Polymer Kit, Vector Laboratories, Newark, CA, USA) for 30 minutes using a humid or moist chamber during incubation steps. 3-3’- diaminobenzidine was used to detect antigen-antibody complex, counter-stained with Harris hematoxylin. For the negative control, the primary antibody was omitted and substituted for phosphate buffered saline. Quantitative analysis was made from 10 fields of middle region of the sockets captured and registered at 100× magnification using the immersion objective (Carl Zeiss Jena GmbH, Jena, Germany) of each animal. For quantification, a 120-point grid was built on ImageJ (Version 1.51, National Institutes of Health, Bethesda, Maryland, USA) to be superimposed on each immunostaining positive cell at intersection points. The results were normalized by the area density (%) of each target, considering 120 points as 100% of the area. The obtained data were statistically analyzed.

## Biochemical analysis

Calcium and phosphate concentrations were determined by endpoint colorimetric methods based on their reaction with purple phthalein in an alkaline medium that formed a violet complex that was measured at 570 nm. Inorganic phosphate concentration was obtained by endpoint ultraviolet photometry, in which its reaction with ammonium heptamolybdate in a highly acidic medium produces a phophomolybdate complex with a 340-nm absorbance that is proportional to the phosphate concentration in the sample. Plasma calcium and phosphate concentrations were expressed in mg/dL. Alkaline phosphatase (ALP) was measured using an adapted modified colorimetric method. Enzymatic activity was determined at 37^o^C in glycine buffer (pH 9.4), containing 2.0 mmol/L of the p-nitrophenyl phosphate substrate (p-NPP, Sigma, St. Louis, EUA) and 2.5 mmol/L of magnesium chloride (MgCl2, Sigma, St. Louis, EUA). The formation of p-nitrophenil was calorimetrically detected at 405 nm using an extinction molar coefficient of 18.000 M^-1^ cm^-1^. The formation of ALP was expressed in International Units (U/L), in which one unit corresponds to the amount of enzyme required to catalyze the transformation of 1 µmol/L of substrate per minute per liter of plasma.

A modified and adapted colorimetric method was used for the total serum TRAP biochemical analysis.^33^ Briefly, samples were subjected to a sodium acetate buffer (pH 5.8) with 5 mmol/L of the p-nitrophenyl phosphate substrate (p-NPP, Sigma, St. Louis, USA) in 50 mM of sodium tartrate and 1 mmol/L of p-hidroxymercuribenzoic acid (p-HMB, Sigma, St. Louis, USA) at 37^o^C. The formation of p-nitrophenyl formation (p-NP) was calorimetrically determined at 405 nm using a molar extinction coefficient of 18.000 M^-1^ cm^-1^. The residual enzymatic activity of total TRAP was expressed in U/L.

## Statistical analysis

The data from the quantitative analyses were assessed for normal distribution using by the Shapiro Wilk test. Statistical analysis was conducted using the Kruskal-Wallis test, followed by the Dunn test for multiple comparisons with a 5% significance level. The statistical analyses were performed on GraphPad Prism 7.0 (GraphPad Software Inc., San Diego, USA).

## Results

### µCT analysis

**Figure 1:**
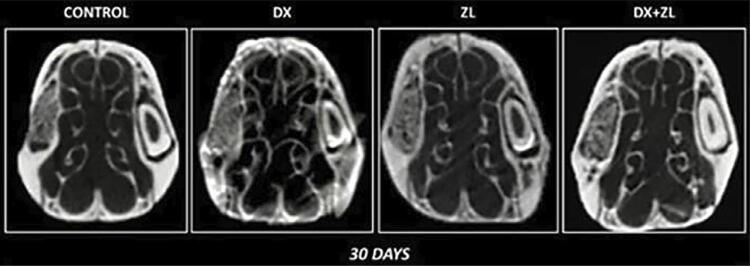
Differential bone formation across groups over 30-day periods. DX significant decreased BV/TV at 30 days when compared to the C (control), ZL, and DX+ZL groups (p<0.05). Significant reductions at 30 days in Tb.Th in the DX group when compared to the C, ZL and DX+ZL groups (p<0.05). No significant differences were observed for all parameters at seven days and in Tb.N and Tb.Sp at 30 days to other groups. 2D images obtained from μCT analysis for socket healing of C, DX, ZL, and DX+ZL groups.

Significant decreased values of BV/TV occurred only at 30 days in DX (72.44±8.58) when compared to C (89.37±2.68), ZL (95.66±1.98), and DX+ZL (87.62±6.09) (p<0.05). Additionally, also at 30 days, significant reductions in Tb.Th occurred in DX (0.33±0.01) when compared to C (0.61±0.09), ZL (0.54±0.06), and DX+ZL (0.51±0.46) (p<0.05). No significant differences occurred for all parameters at 7 days, and in Tb.N and Tb.Sp at 30 days (p>0.05) (Figure 1 Table 1).

### Histopathological findings

**Figure 2:**
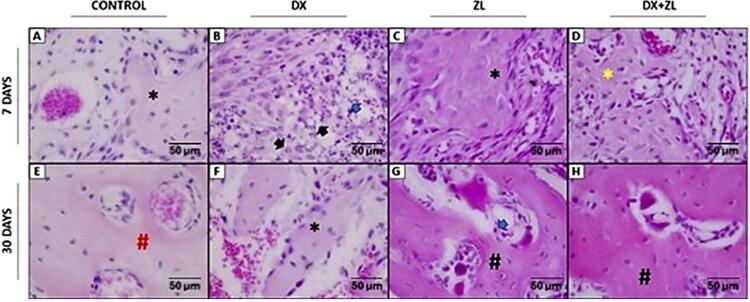
Histopathology of all groups in alveolar socket seven and 30 days after tooth extraction. Seven days – Primary trabeculae in group C (*), whereas foci of neutrophilic leukocytes were observed in granulation tissue of group DX (blue arrow) and eventual pseudoxanthomatous cells (black arrows). Irregular primary trabeculae were noted in ZL (*) and mature remodeling bone in DX+ZL (yellow*). 30 days – Group C showed mature bone (red #). In DX, primary trabeculae could still be seen (*). Bone trabeculae with eventual empty lacunae (#) and foci of leukocytes (blue arrow) were observed in ZL group and mature bone (#) in DX+ZL (HE; original magnification 100x).

On day seven, the dental sockets of the C animals mainly contained highly vascularized granulation tissue with a few mononuclear leukocytes. The new bone formation in the socket walls consisted of immature trabeculae (Figure 2A). The DX animals also showed immature bone trabeculae at the periphery of the sockets. However, a more pronounced inflammatory infiltrate occurred within the granulation tissue comprising mononuclear leukocytes and neutrophils and some pseudoxanthomatous macrophages (Figure 2B). In contrast, the sockets in ZL had granulation tissue showing areas of intense collagen deposition by numerous fibroblasts, along with foci of blood clot. New bone formation also originated from the socket walls, showing immature trabeculae containing large osteocytes. These trabeculae showed irregular osteogenic activity with numerous entrapped osteocytes surrounded by a highly vascularized connective tissue (Figure 2C). Interestingly, DX+ZL, which received both medications, showed thin and irregularly shaped maturing trabeculae with numerous osteocytes under a more uniformly distributed within the sockets. Vascularized connective tissue occurred amidst the trabeculae (Figure 2D).

After 30 days, the sockets in C had fully healed, being filled with mature bone trabeculae mostly covered by lining cells surrounded by loose connective tissue. Some areas also underwent remodeling (Figure 2E). In contrast, the sockets of animals in DX still contained thin bone trabeculae, most of which appeared to be maturing. These trabeculae had irregular shapes and osteoblast activity, showing loose connective tissue (Figure 2F). Animals treated with ZL showed sockets repaired by mature bone trabeculae that resembled those in C. However, they also showed areas of non-viable bone with no osteocytes within the lacunae or osteocytes in karyorrhexis. Focal concentration of mononuclear leukocytes occurred throughout the medullar spaces, as did rounded and non-adhered osteoclasts (Figure 2G). At that time, in which animals received ZL and DX, the healed sockets showed regular and mature trabeculae. The trabeculae were undergoing remodeling and were surrounded by loose connective tissue with mononuclear leukocytes throughout the medullar spaces. Non-adhered osteoclasts also occurred (Figure 2H).

### Histomorphometry

After quantifying the histological parameters, this study analyzed the normal distribution of data by the Shapiro-Wilk test, using the Kruskal-Wallis test followed by Dunn’s test for multiple comparisons. This research set the level of significance of these analyses at 5% (p<0.05). At day seven, DX (19.69±9.40) showed a significantly higher area density of fibroblasts than DX+ZL (5.50±3.30). On the other hand, blood vessels significantly increased in DX+ZL (33.50±16.93) than in DX (7.85±10.17). At day 30, area density of osteoblasts and non-adhered osteoclasts were higher in ZL than in DX (2.81±2.57) and DX+ZL (2.48±2.05). In the same period, mononuclear leukocytes showed higher area density in ZL (2.50±2.70) than in DX (0.29±0.66) and DX+ZL (0.94±2.20). The other parameters in both periods showed no statistical differences (Table 2).

**Table 2:** Means and standard deviations obtained from the quantification of histological parameters at a dosage of 50 μg/kg of ZL.

Groups e periods/ Parameters	C 7 days	C 30 days	DX 7 days	DX 30 days	ZL 7 days	ZL 30 days	DX+ZL 7 days	DX+ZL 30 days
Osteoblasts	2.33±3.22^ab^	1.70±1.36^ab^	2.95±6.02^ab^	2.81±2.57^b^	4.63 ± 5.10^ab^	9.16±5.52^a^	4.80±3.22^ab^	2.48±2.05^b^
Adhered osteoclasts	0.08± 0.27^ab^	0.03±0.18^ab^	0±0^ab^	0±0^ab^	0.23±0.43^ab^	0.20±0.41^ab^	0±0^ab^	0.20±0.49^ab^
Detached osteoclasts	0.04±0.28^ab^	0.06±0.25^ab^	0±0^ab^	0±0^b^	0.10±0.40^ab^	1.10±1.02^a^	0.30±0.67^ab^	0.16±0.42^b^
Fibroblasts	9.33±5.10^ab^	2.96±2.52^ab^	19.69±9.40^a^	5.07±4.02^ab^	8.90±6.42^ab^	6.25±5.11^ab^	5.50±3.30^b^	4.20±4.90^ab^
Blood vessels	14.77±15.16^ab^	18.00±13.93^ab^	7.85±10.17^b^	20.96±15.90^ab^	11.83±8.61^ab^	11.74±12.47^ab^	33.50±16.93^a^	11.96±11.52^ab^
Bone matrix	23.29±25.04^ab^	59.07 ±24.13^ab^	12.3 ±19.91^ab^	41;89±22.52^ab^	30.33±29.01^ab^	35.31±26.68^ab^	51.04±30.63^ab^	51.04±30.63^ab^
Collagen fibers	61.90±29.23^ab^	33.57±21.27^ab^	71.85±22.80^ab^	37.41±21.1^ab^	48.10±27.72^ab^	30.80±26.01^ab^	40.20±17.71^ab^	42.02±29.45^ab^
Inflammatory infiltrate	1.29±1.33^ab^	1.88±2.19^ab^	2.20±3.50^ab^	0.55±0.97^ab^	1.36±2.22^ab^	5.90±4.85^ab^	0.70±1.05^ab^	1.84±3.27^ab^
Mononuclear leukocytes	0.27±0.53^ab^	0.90±1.62^ab^	0.50±1.79^ab^	0.29±0.66^b^	0.46±1.04^ab^	2.50±2.70^a^	0.10±0.3^ab^	0.94±2.20^b^
Polymorphonuclear leukocytes	0.97±1.17^ab^	1.00±1.28^ab^	1.70±1.97^ab^	0.25±0.44^ab^	0.86±1.57^ab^	0.75±0.85^ab^	0.50±0.70^ab^	0.94±2.20^ab^

*Different letters indicate statistically significant differences between groups considering the same parameters within the same periods (p≤0.05). (C=control, DX=dexamethasone, ZL=zoledronate, ZL + DX=zoledronate + dexamethasone, BV/TV=bone volume fraction, Tb.Th=trabecular thickness, Tb.Sp=trabecular spacing, Tb.N=trabecular number).

### Immunohistochemistry analysis

**Figure 3:**
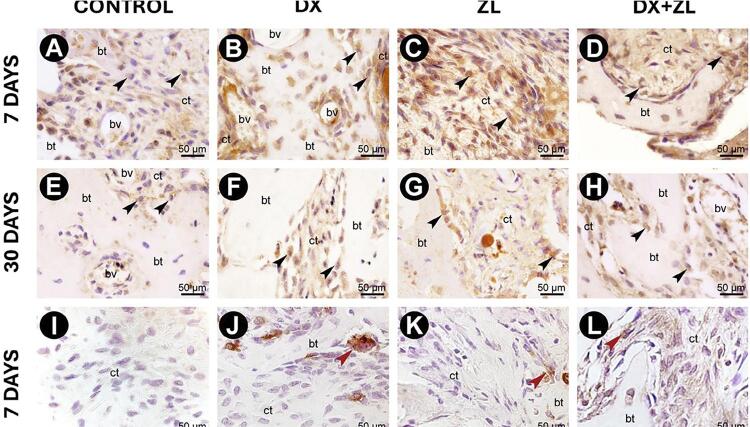
Immunostaining for Runx-2 and TRAP. Runx-2+ cells were predominantly found in the cytoplasm of mesenchymal cells, showing a homogeneous pattern in the control (A) and DX groups (B), and focal distribution in the ZL (C) and DX + ZL (D) groups at seven days. After 30 days, Runx-2+ cells were focal and mostly distributed along the bone trabeculae in the control group (E), DX group (F), and ZL group (G). Notably, the DX+ZL group (H) showed intense Runx-2 positivity. TRAP+ cells were sparsely detected in the control (I) and DX groups (J) and showed a focal distribution in the ZL (K) and DX + ZL (L) groups at seven days. After 30 days, TRAP+ cells showed a similar presence among osteoclasts in the control (M), DX (N), ZL (O), and DX+ZL groups (P). Black arrows indicate TRAP-positive cells and white arrows indicate Runx-2-positive cells (original magnification 100x)

Runx-2^+^ cells predominantly occurred in the cytoplasm of mesenchymal cells, showing a homogeneous pattern in C (Figure 3A) and DX (Figure 3B), unlike ZL and DX+ZL at seven days, which showed focal distribution (Figures 3C and D, respectively). At day seven, significant increased area occupied by Runx-2+ cells occurred in C (4.85±5.75) when compared with ZL (0.85±1.46) and DX+ZL (1.07±1.68) (Table 2). After 30 days, Runx-2^+^ cells were focal and mostly distributed along the bone trabeculae in C (Figure 3E), DX (Figure 3F), and ZL (Figure 3G). Notably, DX+ZL showed intense Runx-2 positivity (Figure 3H). At day 30, C showed significantly more Runx-2^+^ cells than ZL (1.11±1.36) (Table 2).

C and DX showed sparse TRAP^+^ cells at seven days (Figure 3I and J, respectively). These cells showed a focal distribution in ZL (K) and DX+ZL (Figure 3K and L, respectively) without statistical significance. After 30 days, TRAP^+^ cells showed a visually similar intensity and morphology in osteoclasts in all evaluated groups (Figure 3M, N, O, and P). Despite significant differences in TRAP^+^ cells at day 30, C showed a greater area density occupied by TRAP^+^ cells (1.8±2.08) than DX (0.83±0.28) and DX+ZL (0.31±0.79). ZL (1.84±2.39) showed a significant increase in relation to C (1.8±2.08) and DX+ZL (0.31±0.79) (Table 2).

### Biochemistry

The biochemical analysis only considered the 30-day moment to better understand the final and cumulative process. DX (10.51±1.75) showed significantly higher calcium serum levels than ZL (7.36±1.03) and DX+ZL (7.72±1.40). and significantly higher phosphate (7.60±1.21) than ZL (5.26±1.33). Animals in DX (3.61±1.25) also showed higher total serum TRAP levels than those in ZL (2.1±0.19) and DX+ZL (2.16±0.45). On the other hand, ALP serum showed no significant differences (Table 3).

**Table 3:** Means and standard deviations obtained from the quantification of the area density occupied by TRAP and Runx-2 positive cells.

Group	Period	TRAP	Runx-2
Control	7 Days	0.17±0.6^ab^	4.85±5.75^a^
DX		0.59±1.55^ab^	1.95±2.64^ab^
ZL		0.27±0.80^ab^	0.84±1.46^b^
DX+ZL		0.33±0.79^ab^	1.07±1.68^b^
Control	30 Days	1.8±2.08^a^	0.75±1.25^b^
DX		0.83±0.28^bc^	0.76±1.04^ab^
ZL		1.84±2.39^bc^	1.11±1.36^a^
DX+ZL		0.31±0.79^b^	1.5±1.43^ab^

*Different letters indicate significant differences between groups considering the same time point (p<0.05). (C=control, DX=dexamethasone, ZL=zoledronate, ZL + DX=zoledronate + dexamethasone, BV/TV=bone volume fraction, Tb.Th=trabecular thickness, Tb.Sp=trabecular spacing, Tb.N=trabecular number).

## Discussion

The association of DX with high doses of ZL can minimize the acute phase response caused by ZL,^34^ and usually configures a combination for patients receiving treatment for malignant diseases such as multiple myeloma.^33^ Consensus states that high doses of ZL are related to the development of MRONJ^3^, furthering MRONJ onset (especially in experimental models).^15,35,36^ Therefore, low doses of ZL remain poorly explored, although they are indicated to control several clinical conditions, such as osteopenic/osteoporotic conditions^37^ and lung cancer bone metastasis.^38^ Zoledronate shows higher potency than other NBPs,^39^ demanding attention from clinicians if dental procedures are required even in low doses, especially those that involve bone manipulation.^3^ Thus, it is essential to understand the effects of low doses of ZL and DX alone or in combination on post-extraction dental socket repair to improve treatment outcomes and reduce treatment-related side effects. Our results showed distinct roles for these agents separately and combined, denying its null hypothesis.

The selection of any drug dosages and protocol for *in vivo* models is inherently challenging due to the absence of standardized protocols, and ZL is no exception. This study determined its considered low-dose for ZL of 0.05 mg/kg/week via IP administration by meticulously reviewing the literature.^26,40^ Also, our preliminary pilot dose-response study — which evaluated 0.25, and 0.5 mg/kg IP ZL in C57Bl/6J mice — showed that they induced osteonecrosis-like lesions (data not available).

Considering the enhanced bioavailability of IP administration and in accordance with the published dosage ranges, we opted for 0.05 mg/kg/week IP as a low-dose regimen. For DX, the 5-mg/kg/week dose stemmed from Bi, et al.^21^ (2010), which established its efficacy in enhancing zoledronate-associated osteonecrosis of the jaws in male C57BL/6 mice.

Our results show that, post-tooth extraction bone socket repair, DX primarily decreased bone volume and trabecular thickness. Additionally, it was associated with increased levels of circulating calcium, phosphate, and TRAP. Conversely, at the histological level, ZL showed the poorest ability to activate and recruit RUNX-2 and TRAP-positive cells.

The decrease in bone volume and trabeculae and the increased calcium, phosphate, and TRAP levels in DX directly evince imbalances in bone remodeling processes, decreasing bone density and structural integrity. However, DX and C showed no significant differences. Moreover, we highlight the ZL-treated animals alone or combined with DX as they evinced the nBP action on bone metabolism. Although DX has shown positive results in some bone conditions by promoting mesenchymal stem cell differentiation at very low concentrations,^30^ high doses of it suppress osteoblast proliferation and lead to poor outcomes.^41^ Therefore, efforts have aimed to find the optimal dosage and suitable biomaterials to enhance outcomes in bone-related diseases. Controversially, GCs impact calcium metabolism as they can increase calcium excretion from the kidneys and inhibit calcium absorption from the gut, leading to further bone loss.^42^ In line with this, we observed significantly higher levels of calcium and phosphate in DX than in the experimental group, which could have contributed to the impaired healing of the sockets.

The complex process of bone regeneration involves various cells and molecules acting individually or collectively, and histological studies are essential to enhance our understanding of important aspects in this field.^43^ Our interesting histopathological results evinced varying features in the alveolar sockets after extraction according to treatments. Histopathological examination also showed a noticeable delay in socket repair in DX animals than in those in the other groups, particularly regarding bone matrix aspect, which remained clearly immature even after 30 days post-extraction. The significant increased area density of fibroblasts at the beginning of the repair (day seven) and decreased blood vessels in DX when compared to DX+ZL already indicated delayed repair. GCs can alter the lifespan of osteoclasts and inhibit their apoptosis induced by NBPs.^14^ Moreover, GCs can also induce the apoptosis of lymphocytes^10^ and osteocytes, disrupting bone vasculature and significantly decreasing hydraulic support. This alteration affects the mechanosensitive function of osteocytes since the canalicular fluid is directly connected to vascular space,^16^ impacting bone remodeling. Interestingly, when the animals received a concomitant treatment with DX and ZL, they tended to show improved histological bone formation.

Specially, the ZL-treated group showed delayed healing that resembled osteonecrosis of the jaws-like lesions, showing bone trabeculae most of the lacunae of which lacked osteocytes and a greater area density of focal mononuclear leukocytes at day 30 than DX and DX+ZL. Nonetheless, the morphology of the osteoclasts in the treated animals both in the healing phase and at final periods evinced the effects of ZL. In most cases, the osteoclasts showed a round-shaped and a large size, failing to adhere to the bone surface, indicating a disruption in their ability to form the ruffled border.^24,41^ The significant increased area density of these cells in ZL at day 30 confirmed this.

Interestingly, despite most osteoclasts failed to effectively engage in bone resorption, they showed strong TRAP positivity in ZL. As a result, a significantly increased density of TRAP^+^ cells occurred only at day 30 in the healing sockets of ZL when compared to DX+ZL . More importantly, DX+ZL show a lower positivity of TRAP+ cells than C. These findings (in accordance with up-expression of serum TRAP) showed that DX and ZL act by different pathways in bone healing. Such correlation indicates that the overall effectiveness of ZL on skeleton metabolism^44^ operates by a different mechanism than bone healing at a specific site that requires new bone formation, maturation, and remodeling.

Thus, it seems that the opposing mechanisms of DX and ZL on bone cells possible act as a modulatory and balanced environment for bone formation,^14^ as per an *in vitro* study in which GCs prevented the apoptosis of osteoclasts by NBPs by inhibiting caspase 3 (mediated by the GC receptor). Using the RU486 (mifepristone) antagonist could reverse this effect. On the other hand, BPs prevented the apoptosis of osteoblasts by GCs.^14^ Although Runx-2 immunolabeling increased in C animals when compared to those in ZL and DX+ZL during the early stages of healing, the comparison between the experimental groups showed no significant differences. However, Runx2-expressing osteoprogenitors form osteogenic condensations,^45^ suggesting a distinct pattern in groups that receive isolated or concomitant treatments.

However, careful attention must be paid when it comes to animal models and protocols, either with low or high doses of ZL, which can lead the reader to make equivocal comparisons. Similar drug dosage and protocols can obtain different results, as per those in Sonis, et al.^46^ (2009). Young male Sprague-Dawley rats were treated with one, two, or three doses of 0.075 mg/Kg of ZL that were subcutaneously administered alone or combined with 1mg/Kg of DX once a week one day after upper or lower molars extractions. After 28 days of the experiment, most animals that received combined ZL and DX showed non-healed extraction sites, which the authors considered MRONJ. Therefore, Hokugo, et al.^47^ (2010), questioning the lack of mechanism linking the combined use of ZL and DX with the development of MRONJ, decided to induced vitamin D insufficiency in male Sprague-Dawley rats treated with intravenous 0.035mg/Kg of ZL every two weeks during a three-week period, extracting all left maxillary molars. After two weeks, they found MRONJ onset in the ZL treated animals.

This requires considering some points: the use of rats, the extraction site and number of extracted teeth, systemic condition, and drugs protocol. Induction of MRONJ in mice is not as simple as it seems because of the innumerous protocols in the literature and the apparent animal resistance. Kobayashi, et al.^48^ (2010) described the events ZL caused after the extraction of the first right upper molar on the socket and soft tissues healing in young male C57Bl/6J mice, attesting that it caused no osteonecrosis of the jaws.^48^They used 0.25 mg/Kg/day, a dosage almost 10 times higher than that in Hokugo^47^ for seven consecutive days before tooth extraction and for four days after it, in which the animals were euthanized the day after the last ZL dosage. Even older mice require higher doses than those used in rats to induce MRONJ. Biguetti, et al.^24^ (2019) and Mahmoud, et al.^28^ (2021), characterizing dental socket repair in senescence female mice and investigating the effects of 5-lipoxigenase in dental socket repair with or without ZL, respectively, used senescent female 129/Sv mice treated with 0.25 mg/Kg of ZL via IP once a week for four weeks before central incisor tooth extraction, confirming the development of osteonecrosis of the jaws-like lesions. Still, the considered low dose disrupted dental socket repair, as in this study.

To minimize the diverse effects of systemic variables such as gonadal hormones and age-related physiological changes, this study utilized a cohort of young male C57BL/6J mice. However, this methodological approach offers certain limitations. First, the exclusive use of this specific murine population constrains the external validity of our findings, particularly concerning clinically relevant demographics such as aging individuals and females, who show a markedly increased susceptibility to osteoporosis and related skeletal pathologies. Future studies incorporating these diverse demographic subgroups are essential to enable comparative analyses and elucidate potential differences in post-extraction alveolar bone healing under standardized experimental conditions. Second, while the murine model provides a high degree of experimental control, its translational applicability to human physiology and clinical practice remains limited. Therefore, further research employing human cell-based *in vitro* models, alongside clinical trials incorporating biomechanical assessments of bone integrity, is critical to bridging the translational gap between murine and human responses.

## Conclusion

Overall, our results show that combining low doses of ZL with a DX treatment in male young mice enhanced the healing of post-extraction sockets compared to each treatment alone. However, further investigations are warranted to explain the underlying mechanisms in a more controlled manner. This approach may serve as an alternative to improve post-tooth extraction healing in individuals undergoing long-term treatment with GCs.

**Table 4:** Means and standard deviations obtained from biochemical analyses of bone metabolism markers, calcium (mg/dL), phosphate (mg/dL), ALP (U/L), and TRAP (U/L).

Biochemical Markers	C	ZL	DX	DX+ZL
Calcium	8.31±1.67^ab^	7.36±1.03^a^	10.51±1.75^b^	7.72±1.40^b^
Phosphate	6.93±0.74^a^	5.26±1.33^a^	7.60±1.21^b^	6.54±1.50^a^
ALP	2.20±0.38^a^	2.11±0.20^a^	2.36±0.18^a^	2.26±0.23^a^
TRAP	2.95±1.21^ab^	2.1±0.19^b^	3.61±1.25^a^	2.16±0.45^b^

*Different letters indicate significant differences between groups (p<0.05). (C=control, DX=dexamethasone, ZL=zoledronate, ZL + DX=zoledronate + dexamethasone, BV/TV=bone volume fraction, Tb.Th=trabecular thickness, Tb.Sp=trabecular spacing, Tb.N=trabecular number).
